# Genotype–phenotype correlations in WHIM syndrome: a systematic characterization of *CXCR4*^WHIM^ variants

**DOI:** 10.1038/s41435-022-00181-9

**Published:** 2022-09-12

**Authors:** Katarina Zmajkovicova, Sumit Pawar, Sabine Maier-Munsa, Barbara Maierhofer, Ivana Wiest, Renato Skerlj, Arthur G. Taveras, Adriana Badarau

**Affiliations:** 1 X4 Pharmaceuticals (Austria) GmbH, Vienna, Austria; 2 X4 Pharmaceuticals Inc, Boston, MA USA

**Keywords:** Haplotypes, Chemokines

## Abstract

Warts, hypogammaglobulinemia, infections, myelokathexis (WHIM) syndrome is a rare primary immunodeficiency predominantly caused by heterozygous gain-of-function mutations in CXCR4 C-terminus. We assessed genotype–phenotype correlations for known pathogenic CXCR4 variants and in vitro response of each variant to mavorixafor, an investigational CXCR4 antagonist. We used cell-based assays to analyze CXCL12-induced receptor trafficking and downstream signaling of 14 pathogenic CXCR4 variants previously identified in patients with WHIM syndrome. All CXCR4 variants displayed impaired receptor trafficking, hyperactive downstream signaling, and enhanced chemotaxis in response to CXCL12. Mavorixafor inhibited CXCL12-dependent signaling and hyperactivation in cells harboring CXCR4^WHIM^ mutations. A strong correlation was found between CXCR4 internalization defect and severity of blood leukocytopenias and infection susceptibility, and between AKT activation and immunoglobulin A level and CD4^+^ T-cell counts. This study is the first to show WHIM syndrome clinical phenotype variability as a function of both *CXCR4*^WHIM^ genotype diversity and associated functional dysregulation. Our findings suggest that CXCR4 internalization may be used to assess the pathogenicity of *CXCR4* variants in vitro and also as a potential WHIM-related disease biomarker. The investigational CXCR4 antagonist mavorixafor inhibited CXCL12-dependent signaling in all tested CXCR4-variant cell lines at clinically relevant concentrations.

## Introduction

Warts, hypogammaglobulinemia, infections, and myelokathexis (WHIM) syndrome is a rare, autosomal-dominant primary immunodeficiency disease (PID, OMIM: #193670) [1, 2]. It manifests with an array of symptoms with variable clinical presentations, including panleukopenia, increased susceptibility to recurrent bacterial infections and human papillomaviruses (HPVs), congenital heart defects, and increased risk of malignancy [2, 3]. WHIM syndrome pathogenesis is causally linked to a variety of heterozygous gain-of-function (GOF) mutations in the C-terminus of the C-X-C chemokine receptor 4 (CXCR4), a master regulator of immune cell trafficking and homeostasis, causing desensitization defects, hyperactivation of downstream signaling (G-protein signaling, calcium mobilization, ERK/AKT activation), and retention of leukocytes in the bone marrow [3–11]. *CXCR4* GOF somatic mutations have also been reported in patients with Waldenström’s macroglobulinemia (WM), a rare B-cell lymphoma [2, 12]. In clinical studies of patients with WM, segregation between nonsense and frameshift *CXCR4* mutations was observed in response to ibrutinib treatment and overall survival [13, 14]. However, a comprehensive side-by-side characterization of *CXCR4* variants, to delineate whether potential functional differences of the individual *CXCR4* mutations could explain the variable phenotypic presentation of patients with WHIM syndrome, was lacking. Such analysis would also be informative to identify potential biomarkers for WHIM syndrome diagnosis and to reclassify *CXCR4* variants of uncertain significance.

In this study, we aimed to connect the functional profiles of a large spectrum of *CXCR4*^WHIM^ mutations to phenotypic manifestations in patients with WHIM syndrome. To this end, we generated and tested cellular models of WHIM syndrome to (i) compare side-by-side the in vitro functional activity of pathogenic CXCR4^WHIM^ variants; (ii) evaluate each variant’s in vitro response to mavorixafor, a CXCR4 antagonist currently in clinical trials for the treatment of patients with WHIM syndrome and WM carrying *CXCR4* GOF mutations [15]; and (iii) explore genotype-function-phenotype correlations in patients with WHIM syndrome.

## Methods

### Internalization assay

Stable K562 clones expressing CXCR4 (1 × 10^5^ cells/well) were seeded in 96-well plates and serum starved for 24 h. Cells were then resuspended in warm incubation buffer (Hanks’ Balanced Salt Solution [HBSS] with Ca^2+^ and Mg^2+^ + 0.5% BSA + 20 mM HEPES buffer pH 7.4) and stimulated with C-X-C chemokine ligand 12 (CXCL12) for 45 min or 4 h at 37 °C, 5% CO_2_. After incubation, the cells were washed twice with cold incubation buffer and then stained with anti-CXCR4 12G5-APC monoclonal antibody (Product code 555976, BD Biosciences; 1:20 dilution in incubation buffer) for 20 min at 4 °C. After washing and resuspending in flow buffer (HBSS with Ca^2+^ and Mg^2+^ + 0.1% BSA + 20 mM HEPES buffer pH 7.4), the samples were measured via flow cytometry (CytoFLEX, Beckman Coulter Diagnostics, Brea, CA, USA) and analyzed using FCS Express™ flow cytometry software (De Novo, Pasadena, California, USA). Cells were gated based on the forward and side scatter (FSC and SSC, respectively) and isotype control, and the mean fluorescent intensity (MFI) of the CXCR4+ population was used in subsequent analysis.

### Chemotaxis assay

K562 cells (2.5 × 10^6^ cells in 250 µl Ingenio Electroporation Solution per 0.4-cm cuvette) were transiently transfected by electroporation (Gene Pulser Xcell^TM^ System, Bio-Rad Hercules, California, USA) with the corresponding pcDNA3.1_CXCR4 plasmid (25 µg), using an exponential decay protocol (250 V, 1000 µF), and then immediately transferred to 6-well plates containing 2.25 ml pre-equilibrated culture medium. Following 24-h incubation, transfected cells were serum starved in IMDM supplemented with 0.5% BSA, and after another 24 h, chemotaxis was performed using 6.5-mm transwell plates with 8.0-µm pore size (Corning Incorporated, Corning, New York, USA). Prior to chemotaxis, cells were stained with 500 nM Calcein AM Viability Dye (Thermo Fisher Scientific, Waltham, Massachusetts, USA) in IMDM supplemented with 0.5% BSA for 15 min at room temperature in the dark. Aliquots of 2 × 10^5^ cells in 100 µl IMDM supplemented with 0.5% BSA and 20 µg/ml fibronectin (Sigma-Aldrich, St Louis, Missouri, USA) were added to the plate inserts, and 600 µl buffer with or without indicated CXCL12 concentrations were added to the bottom wells. Cells were allowed to migrate in response to CXCL12 at 37 °C and 5% CO_2_ for 4 h. For Jurkat cells, the chemotaxis was performed using 6.5 mm transwell plates with 3.0 µm pore size (Corning Incorporated, Corning, New York, USA). Cells were harvested in assay buffer (RPMI + 10 mM HEPES + L-Glutamine + 0.5% BSA) and stained with 500 nM Calcein AM Viability Dye for 15 min at RT in the dark. 1 × 10^6^ cells in 100 µl was added in the insert and allowed to migrate toward CXCL12 in the bottom wells for 2 h. After removal of the plate inserts, the migrated cells were centrifuged and resuspended in Dulbecco’s phosphate buffered saline containing flow cytometry counting beads (Precision Count Beads™, BioLegend, San Diego, California, USA). Both migrated cells and counting beads were counted by flow cytometry (CytoFLEX, Beckman Coulter Diagnostics, Brea, California, USA). Data were analyzed using FCS Express software, and the total number of migrated cells was calculated, according to the counted and total number of beads present in the sample.

### Flow cytometry—phosphoflow

Stable K562 clones expressing CXCR4 or Jurkat cells were seeded in starvation medium in 96-well plates overnight. After treatment, the cells were fixed with fixation solution (eBioscience, Thermo Fisher Scientific, Waltham, Massachusetts, USA) for 10 min at room temperature, then permeabilized for 5 min with ice-cold methanol on ice followed by a washing step with wash/permeabilization solution (eBioscience, Thermo Fisher Scientific, Waltham, Massachusetts, USA). Cells were then stained with Alexa Fluor 647 Mouse Anti-ERK1/2 (Product code 612593, pT202/pY204, 1/10 dilution, BD Biosciences) and Alexa Fluor 488 Mouse anti-Akt (Product code 560404, pS473, 1/10 dilution, BD Biosciences) for 1 h at room temperature in the dark. Cells were then washed 2 times in wash/permeabilization solution and resuspended in flow buffer (HBSS with Ca^2+^ and Mg^2+^ + 0.1% BSA + 20 mM HEPES pH 7.4). Samples were measured via flow cytometry (CytoFLEX Flow Cytometer) and analyzed in FCS Express software. After gating cells based on the FCS/SSC, MFI of the respective staining was exported for further analysis.

### Ligand binding inhibition assay

Ligand binding inhibition by mavorixafor was measured as previously published [16].

### Cyclic adenosine monophosphate (cAMP) enzyme-linked immunosorbent assay (ELISA)

Production of cAMP was assessed using the cAMP-Screen™ Cyclic AMP Immunoassay System (Applied Biosystems) according to manufacturer’s instructions.

### Statistical analysis

Statistical analysis was performed in Prism. *P* values < 0.05 were considered statistically significant and set as follows: *P < 0.05; **P < 0.01; ***P < 0.001. Unpaired 2-tailed *t* test was used in most cases, while 1 sample 2-tailed *t* test was used when data were normalized to control sample (H0: sample means are equal to 1). The number of independent experiments (n) is stated in each figure legend. Calculation of EC_50_/IC_50_ and E_max_ parameters of CXCL12 or mavorixafor was performed in Prism by log(agonist/antagonist) versus response—variable slope (4 parameters) function. Relationships among in vitro parameters were analyzed using Pearson correlation, while those between in vitro parameters and clinical phenotypes were analyzed using both Pearson and Spearman correlations.

See Supplementary Material for further details on experimental procedures.

## Results

### Analysis of 14 CXCR4^WHIM^ variants reveals internalization defects and hyperactive downstream signaling

**Fig. 1:**
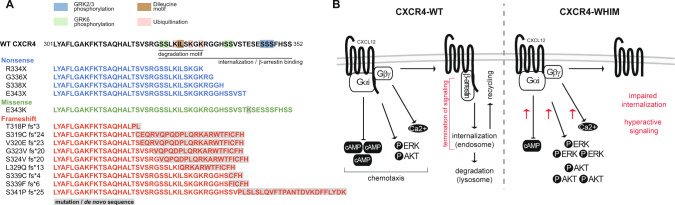
Stucture and signaling of CXCR4^WHIM^ variants. **A** WT CXCR4 sequence (C-terminus from amino-acid 301) with posttranslational modifications and motifs involved in receptor trafficking indicated. C-terminal sequences of CXCR4^WHIM^ variants investigated in the present study. **B** Schematic representation of signaling pathways activated downstream of the CXCR4 receptor and the effect of CXCR4^WHIM^ mutations. AKT protein kinase B, cAMP cyclic adenosine monophosphate, CXCL12 C-X-C chemokine ligand 12, CXCR4 C-X-C chemokine receptor 4, ERK extracellular signal-regulated kinase, fs frameshift, GRK G protein–coupled receptor kinase, WHIM Warts, Hypogammaglobulinemia, Infections, and Myelokathexis, WT wild-type.

Previous reports have disclosed individual CXCR4^WHIM^ mutations [6–9, 11, 17, 18] with impaired desensitization and hyperactive downstream signaling. However, there is no comprehensive characterization of a larger spectrum of CXCR4 variants, performed in parallel in a controlled study, to allow the identification of potential functional similarities and differences amongst the variants, and to better understand the diversity and breadth of WHIM syndrome from a genotype–phenotype perspective. Accordingly, we screened published reports to gather the collection of known pathogenic CXCR4 mutations in patients with WHIM syndrome [2, 3, 6, 8–10, 18–21]. The 14 identified *CXCR4* mutations (4 nonsense, 9 frameshift, 1 missense) span the CXCR4 C-terminal region representing amino-acid residues 318 to 343 (Fig. 1A). Similar to previous studies, we used the K562 cell line (CXCR4 negative) [22] as a model system to express all variants at comparable levels (Fig. S1A) [8, 9, 22]. The established cell lines (or a transient expression system) were then used to characterize the variants in in vitro assays analyzing CXCR4 trafficking, downstream signaling and chemotaxis (Fig. 1B) in response to CXCL12.

**Fig. 2:**
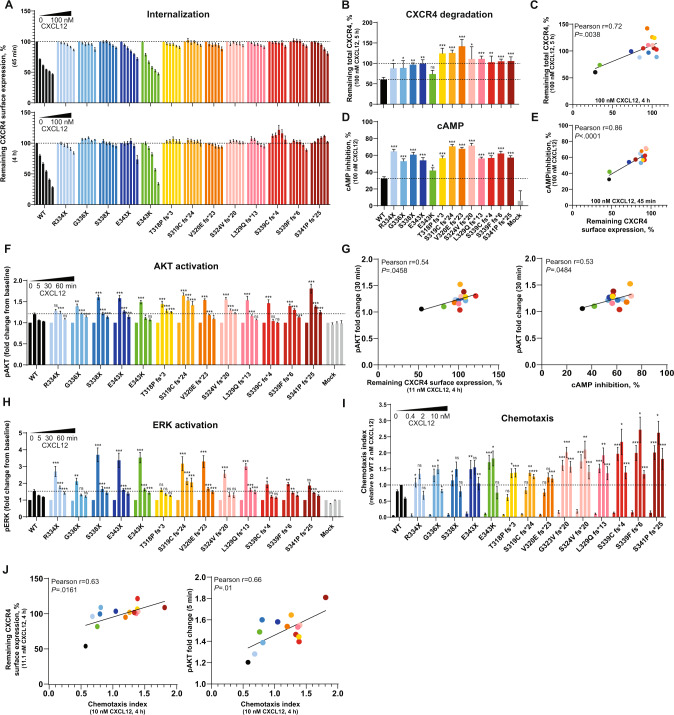
Functional assays in CXCR4^WHIM^ variants. **A** K562 cells with stable CXCR4 expression were stimulated with CXCL12 (vehicle, 1.2 nM, 3.7 nM, 11 nM, 33 nM, 100 nM) for 45 min or 4 h, and the surface expression of CXCR4 was measured by flow cytometry. Values are expressed as % remaining CXCR4 compared to vehicle-treated cells. Values represent mean ± SEM of 4 clonal lines per variant analyzed in 4 independent experiments. **B** K562 cells with stable CXCR4 expression were stimulated with vehicle or 100 nM CXCL12 for 5 h, and the whole-cell lysates were analyzed by western blot to determine total CXCR4 levels. Samples from individual clonal lines were pooled per variant in each experiment. Values represent % of treated sample compared to vehicle control. Mean ± SEM, *n* = 3–4. **C** Plot showing correlation between CXCR4 degradation/CXCR4 internalization. Linear regression was used to analyze the correlation of measured values. **D** K562 cells with stable CXCR4 expression were stimulated with forskolin ± 100 nM CXCL12 for 30 min. cAMP production was measured by ELISA. % Inhibition of cAMP production by CXCL12 was calculated respective to forskolin-only treated cells. Values represent mean ± SEM of 4 clonal lines per variant analyzed in 4 independent experiments. **E** Plots showing correlation between cAMP inhibition/CXCR4 internalization. Linear regression was used to analyze the correlation of measured values. **F** K562 cells with stable CXCR4 expression were stimulated with 10 nM CXCL12 for 5, 30, or 60 min, fixed, and the median fluorescence intensity of p-S473 AKT staining was measured by flow cytometry. Values are expressed as FC compared to unstimulated sample. Mean ± SEM of 4 clonal lines per variant analyzed in 4 independent experiments. **G** Plots showing correlation between p-S473 AKT (30 min)/CXCR4 internalization (left panel) and p-S473 AKT (30 min)/cAMP inhibition (right panel). Linear regression was used to analyze the correlation of measured values. **H** K562 cells with stable CXCR4 expression were stimulated with 10 nM CXCL12 for 5, 30, or 60 min, fixed and the median fluorescence intensity of p-T202/Y204 ERK staining was measured by flow cytometry. Values are expressed as FC compared to unstimulated sample. Mean ± SEM of 4 clonal lines per variant analyzed in 4 independent experiments. **I** K562 cells transiently transfected with indicated CXCR4 constructs were subjected to transwell chemotaxis assay. Cells migrated towards 0.4, 2, 10 nM CXCL12 or medium only for 4 h. The total number of migrated cells was normalized to WT with 2 nM CXCL12 in each assay. Mean ± SEM, *n* = 4–14. **J** Plots showing correlation between CXCR4 internalization/chemotaxis (left panel) and p-S473 AKT (5 min)/chemotaxis (right panel). Linear regression was used to analyze the correlation of measured values. AKT protein kinase B, CXCL12 C-X-C chemokine ligand 12, CXCR4 C-X-C chemokine receptor 4, cAMP cyclic adenosine monophosphate, ELISA enzyme-linked immunosorbent assay, ERK extracellular signal-regulated kinase, FC fold change, fs frameshift, ns not significant, SEM standard error of mean, WT wild-type, WHIM Warts, Hypogammaglobulinemia, Infections, and Myelokathexis.

The CXCR4 receptor internalization defect is one of the main hallmarks of CXCR4^WHIM^ receptors in WHIM syndrome patient cells [5]. All CXCR4^WHIM^ variants exhibited impaired receptor internalization in response to CXCL12 (Figs. 2A, S1B, Table S2). The missense variant E343K was least affected and showed decreased internalization only at lower CXCL12 concentrations at the 4-h timepoint. E343X and S341Pfs*25 variants displayed partial internalization at the highest CXCL12 concentrations tested after 45 min of ligand stimulation. The impaired internalization of CXCR4^WHIM^ variants correlated with decreased receptor degradation (Fig. 2B, C, Table S2) and increased cAMP inhibition (Fig. 2D, E, Table S2). As seen in the internalization assays, the E343K mutation conferred the mildest phenotype in these parameters. Most variants demonstrated a higher amplitude and/or duration of extracellular signal-regulated kinase (ERK) and AKT activation upon stimulation with CXCL12 (Fig. 2F, H, Table S2), with the exception of T318Pfs*3, which had a normal profile of ERK activation kinetics. AKT signaling was consistently prolonged and followed similar trends as CXCR4 internalization and cAMP inhibition (Fig. 2F, G). Chemotaxis towards CXCL12 was increased in CXCR4^WHIM^ variants to different extents in at least one of the tested CXCL12 concentrations. For the more truncated frameshift variants (T318Pfs*3, S319Cfs*24, V320Efs*23), chemotaxis increased only at higher CXCL12 concentrations; other frameshift variants showed enhanced chemotaxis regardless of CXCL12 concentration (Fig. 2I). The CXCL12-induced migration of CXCR4 variants correlated with the extent of receptor internalization as well as with AKT activation amplitude (Fig. 2J), in line with the role of the PI3K/AKT axis in chemotactic responses toward chemokines [23]. In contrast to hyperactive cAMP inhibition and AKT/ERK signaling, Ca^2+^ mobilization was not enhanced in CXCR4^WHIM^-expressing K562 cells compared to K562 control cells expressing wild-type (WT) CXCR4 (E_max_ range, 68–117%; Fig. S1C, Table S2). Taken together, CXCR4^WHIM^ variants are characterized by a general impairment of ligand-induced internalization and degradation of mutated receptor paralleled with hyperactive downstream signaling and increased chemotactic response.

### Cellular Jurkat model of heterozygous and homozygous *CXCR4*^WHIM^ mutations recapitulates the gain-of-function phenotypes found in patients with WHIM syndrome and in K562 cells

Greater than 85% of patients with WHIM syndrome harbor heterozygous *CXCR4* mutations [24]. The most frequently reported mutation is c.1000C>T, which results in a premature C-terminal truncation of the receptor at position R334 (R334X) [24]. Primary cells isolated from patients with the R334X variant display CXCR4 receptor internalization defects and GOF phenotypes in signaling assays [8, 22]. In vitro assays using cell lines with exogeneous overexpression of *CXCR4*^WHIM^ variants can model the GOF cellular phenotypes (Fig. 2 and related literature [8, 9, 17, 22, 25]), but may not entirely represent the situation in patients with WHIM syndrome, who are heterozygous and have one wild-type *CXCR4* allele. Therefore, in addition to the K562 transfected cell lines expressing CXCR4 variants, we also aimed to characterize a cellular model of homozygous and heterozygous R334X CXCR4 in an endogenous locus to better understand the (pathogenic) impact of having one or both alleles mutated. We established the first model recapitulating the heterozygous mutations found in patients with WHIM syndrome using the CRISPR-Cas9 platform. A Jurkat cell line (with endogenous expression of WT *CXCR4*) [26] was edited to harbor the c.1000C>T/R334X mutation in a single allele (clone B2, RX/WT) or in both alleles (clones A3 and B5, RX/RX). As controls, we used the unedited parental Jurkat cell line and Jurkat cells with edited silent mutations (clone C3, WT/WT).

**Fig. 3:**
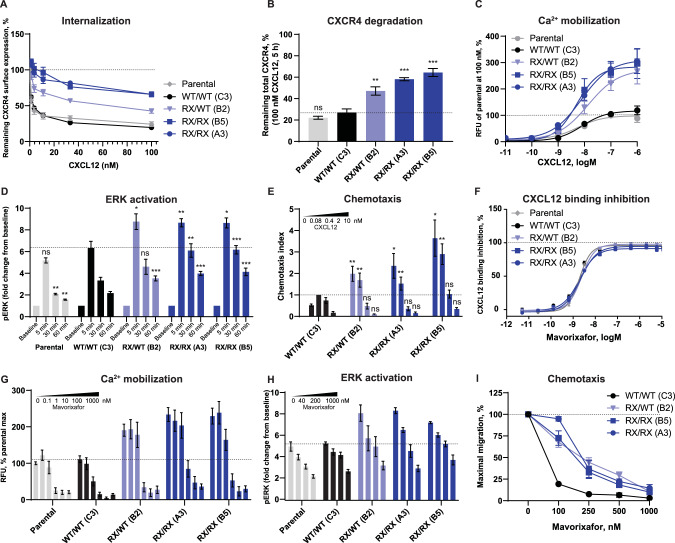
Functional analysis and sensitivity to mavorixafor in Jurkat cells expressing CXCR4^R334X^. **A** Jurkat cells were stimulated with CXCL12 (vehicle, 1.2 nM, 3.7 nM, 11 nM, 33 nM, 100 nM) for 45 min, and the surface expression of CXCR4 was measured by flow cytometry. Values are expressed as % remaining CXCR4 compared to vehicle-treated cells. Values represent mean ± SEM, *n* = 6. **B** Jurkat cells were stimulated with vehicle or 100 nM CXCL12 for 5 h, and the whole-cell lysates were analyzed by western blot to determine total CXCR4 levels. Samples from individual clonal lines were pooled per variant in each experiment. Values represent % of treated sample compared to vehicle control. Mean ± SEM, *n* = 4. **C** Jurkat cells were stimulated with serial dilutions of CXCL12 to measure Ca^2+^ mobilization. RFU measured in parental line at 100 CXCL12 represented 100%. Mean ± SEM, *n* = 4. **D** Jurkat cells were stimulated with 10 nM CXCL12 for 5, 30, or 60 min, fixed and the median fluorescence intensity of p-T202/Y204 ERK staining was measured by flow cytometry. Values are expressed as FC compared to unstimulated sample. Mean ± SEM, *n* = 6–7. **E** Jurkat cells were subjected to transwell chemotaxis assay. Cells migrated towards 0.08, 0.4, 2, 10 nM CXCL12 or medium only for 2 h. The total number of migrated cells was normalized to WT with 0.4 nM CXCL12 in each assay. Mean ± SEM, *n* = 4. **F** Jurkat cells were pretreated with serial dilutions of mavorixafor, incubated with AF647-CXCL12 and subjected to flow cytometry to determine the ligand binding inhibition potency of mavorixafor. Mean ± SEM, *n* = 4. **G** Jurkat cells were preincubated with serial dilutions of mavorixafor and then stimulated with 100 nM CXCL12 to measure Ca^2+^ mobilization. RFU measured in parental line at 100 CXCL12 represented 100%. Mean ± SEM, *n* = 4–10, **H** Profiling of mavorixafor in ERK activation assays. Jurkat cells were preincubated with vehicle, 40 nM, 200 nM, or 1 µM mavorixafor and then stimulated with 10 nM CXCL12 for 5 min. Median fluorescence intensity of p-T202/Y204 ERK staining was measured by flow cytometry. Values are expressed as FC compared to unstimulated cells. SEM, *n* = 6–8. **I** Profiling of mavorixafor in chemotaxis assays. Jurkat cells were preincubated with vehicle, 100 nM, 250 nM, 500 nM, or 1 µM mavorixafor and subjected to migration towards 0.4 nM CXCL12. % maximal migration was determined with respect to the vehicle-pretreated sample. Mean ± SEM, *n* = 3. When statistics are indicated, samples were compared to WT/WT (C3) clone for the respective conditions. CXCR4 C-X-C chemokine receptor 4, CXCL12 C-X-C chemokine ligand 12, ERK extracellular signal-regulated kinase, FC fold change, ns not significant, RFU relative fluorescent unit, RX R334X, SEM standard error of mean, WT wild-type.

Similar to CXCR4-expressing K562 cells, we measured CXCR4 trafficking and signaling responses in the generated R334X Jurkat cell lines. To measure the desensitization response, we treated cells with increasing concentrations of CXCL12 for 45 min. RX/RX cell lines displayed an internalization defect (65% of CXCR4 receptors remaining on the cell surface at 100 nM CXCL12) compared to the parental (24%) and WT lines (20%). The RX/WT-expressing cells had an intermediate phenotype (43%; Fig. 3A). Upon internalization, the CXCR4 receptor is predominantly sorted to lysosomes and degraded [27]. This was the case in cells having WT *CXCR4*, in which the total levels of the receptor decreased by 73–78% after 5-h incubation with 100 nM CXCL12. RX/RX variants showed impaired CXCR4 degradation (36–42% decrease) and the RX/WT cells again displayed intermediate effects (53% decrease, Fig. 3B) in the degradation assay. Next, we analyzed the signaling responses downstream of activated CXCR4. Calcium mobilization in response to CXCL12 was enhanced in cells harboring the R334X mutation, reaching a ± 3-fold higher E_max_ than cells with WT CXCR4 (Fig. 3C, Table S3). ERK activation downstream of CXCR4 reached a higher amplitude (8-fold increase over baseline) and duration after stimulation with CXCL12 in all lines expressing R334X compared to the parental and WT cell lines (sixfold increase; Fig. 3D). Of note, in both signaling readouts, one mutant allele seemed to confer the full GOF phenotype and did not show the intermediate phenotype observed in receptor trafficking assays. Migration of cells toward CXCL12 was significantly enhanced in R334X-expressing cells and displayed an altered concentration-response curve (highest migration at 0.08 nM; Fig. 3E). The two homozygous clones showed different chemotactic responses (A3 was comparable to RX/WT and B5 migrated more than RX/WT) and therefore we were not able to conclude on the differences between heterozygous and homozygous mutations. In summary, we generated the first cellular model that recapitulates both the genetic changes in WHIM syndrome as well as the CXCR4 desensitization defect paralleled by the GOF phenotype in downstream signaling assays.

### Mavorixafor shows comparable activity on CXCR4 WT, heterozygous and homozygous R334X-expressing cells

Mavorixafor is a potent and selective investigational CXCR4 antagonist currently being studied in clinical trials for the treatment of patients with WHIM syndrome (NCT03005327) [15]. We aimed to assess mavorixafor activity in R334X-expressing Jurkat cells, as this model allows us to compare the response to mavorixafor in cells endogenously expressing WT, R334X/WT or R334X/R334X CXCR4 at similar levels and, most importantly, in the R334X/WT cells that model the genotype of patients with WHIM syndrome. Mavorixafor was found to inhibit CXCL12 binding to CXCR4 with 100% efficacy and with nearly identical potency (1.6–2.4 nM; Table S3) across all cell lines (WT/WT, RX/WT, RX/RX; Fig. 3F). Mavorixafor also inhibited CXCL12-stimulated calcium mobilization with comparable potency (Fig. 3G, Table S3); ERK activation (Fig. 3H) in all cell lines tested; and normalized the hyperactive ERK signaling in RX/WT and RX/RX cells at 40 nM and 200 nM concentrations, respectively. Chemotaxis toward CXCL12 was efficiently inhibited by increasing concentrations of mavorixafor in all edited Jurkat cells, with *CXCR4* WT/WT-expressing cells being the most sensitive (Fig. 3I, Table S3). Taken together, we showed that mavorixafor is active in an unbiased manner on a spectrum of CXCR4-related readouts with high efficacy and potency in cells expressing the WT and/or R334X CXCR4 receptor.

### Mavorixafor inhibits CXCL12/CXCR4-dependent signaling in all tested CXCR4^WHIM^ variants

**Fig. 4:**
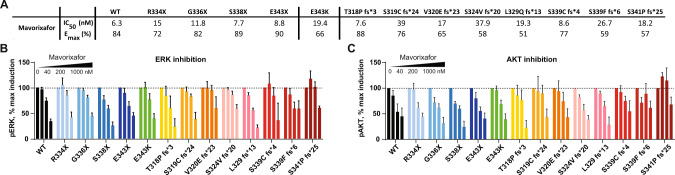
Effects of mavorixafor on CXCR4-CXCL12 signaling in K562 cells expressing CXCR4^WHIM^ variants. **A** E_max_ (% inhibition) and IC_50_ values of mavorixafor determined in Ca^2+^ mobilization assays. K562 cells with stable CXCR4 expression were preincubated with serial dilutions of mavorixafor and then stimulated with 10 nM CXCL12. *n* = 4–17. **B** Profiling of mavorixafor in ERK activation assays. Serum-starved K562 cells with stable CXCR4 expression were preincubated with 40 nM, 200 nM, or 1 µM mavorixafor and then stimulated with 10 nM CXCL12 for 5 min. Median fluorescence intensity of p-T202/Y204 ERK staining was measured by flow cytometry. Values are expressed as % maximal induction in the vehicle-treated cells. Mean ± SEM, *n* = 3–17. **C** Profiling of mavorixafor in AKT activation assay. Serum-starved K562 cells with stable CXCR4 expression were preincubated with 40 nM, 200 nM or 1 µM mavorixafor and then stimulated with 10 nM CXCL12 for 5 min. The median fluorescence intensity of p-S473 AKT staining was measured by flow cytometry. Values are expressed as % maximal induction in the vehicle-treated cells. Mean ± SEM, *n* = 3–14. AKT protein kinase B, CXCR4 C-X-C chemokine receptor 4, CXCL12 C-X-C chemokine ligand 12, E_max_ maximum effect, ERK extracellular signal-regulated kinase, IC_50_ half-maximal inhibitory concentration, SEM standard error of mean.

Mavorixafor is currently being studied in a late-stage clinical trial of patients with WHIM syndrome harboring a spectrum of *CXCR4*^WHIM^ mutations. However, the in vitro activity of mavorixafor has previously only been reported for two pathogenic variants (R334X and E343X) [28]. We aimed to determine the in vitro efficacy and potency of mavorixafor on the whole spectrum of CXCR4^WHIM^ variants expressed in K562 cells. We measured the drug parameters in in vitro CXCR4 downstream signaling assays following CXCL12 stimulation. Mavorixafor was found to inhibit Ca^2+^ mobilization in cells expressing WT CXCR4 (IC_50_ = 6.3 nM; E_max_ = 84%) and in all CXCR4^WHIM^ variants (IC_50_ = 7.6–39 nM; E_max_ = 51–90%; Figs. 4A, S2A). The investigated variants proved to be sensitive to mavorixafor, demonstrating potent CXCR4-dependent signal inhibition using a broad range of CXCL12 concentrations (1–100 nM; Fig. S2B). Mavorixafor also inhibited CXCL12-dependent ERK/AKT activation (Fig. 4B, C) and prevented CXCL12-induced hyperactivation of ERK and AKT in CXCR4^WHIM^ variants (Fig. S2C, D). Taken together, WT CXCR4 and all tested CXCR4^WHIM^ variants were sensitive to pharmacological intervention with mavorixafor at drug concentrations used in clinical trials of patients with WHIM syndrome [15].

### Genotype–phenotype correlations in WHIM syndrome

**Fig. 5:**
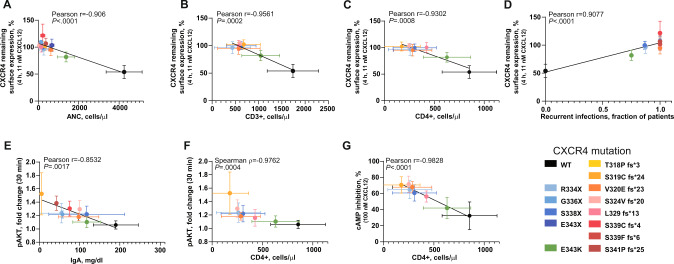
Genotype–phenotype correlations for CXCR4^WHIM^ variants. **A–G** Plots showing correlation between (**A**) ANC levels/CXCR4 internalization, (**B**) CD3^+^ cells/CXCR4 internalization, (**C**) CD4^+^ cells/CXCR4 internalization, (**D**) recurrent infection rates/CXCR4 internalization, (**E**) pAKT/IgA levels, (**F**) pAKT/CD4^+^ cells, and (**G**) cAMP inhibition/CD4^+^ cells. Linear regression was used to analyze the correlation of measured values in (**A–E**, **G**), Spearman correlation was used in (**F**). Values are plotted as mean ± SD. AKT protein kinase B, ANC absolute neutrophil count, cAMP cyclic adenosine monophosphate, CD cluster of differentiation, E_max_ maximum effect, ERK extracellular signal-regulated kinase, IC_50_ half-maximal inhibitory concentration, IgA immunoglobulin A, WT wild-type.

With the in vitro functional parameters of all CXCR4^WHIM^ variants at hand, we interrogated these data for potential correlations with clinical phenotypes reported for patients harboring these mutations (Table S4). We considered quantifiable disease manifestations associated with WHIM syndrome (fraction of affected patients with warts, hypogammaglobulinemia, recurrent infections, and myelokathexis), blood biomarkers (total white blood cell count, subpopulation counts, immunoglobulin [IG] levels) and determined Pearson and Spearman correlation coefficients for these parameters (Table S5). We considered significant only those correlations that reached significance with and without inclusion of the physiological values for WT CXCR4 (Table S5). The CXCR4 internalization defect demonstrated for each CXCR4^WHIM^ variant strongly correlated with the absolute neutrophil count and absolute CD3^+^ and CD4^+^ T-cell count in peripheral blood of patients carrying the respective *CXCR4*^WHIM^ mutation (Figs. 5A–C, S2E). This observation connects the main hallmark of CXCR4^WHIM^ variants, impaired desensitization, with the most penetrant phenotypic clinical manifestations in patients with WHIM syndrome; neutropenia, and lymphopenia. The degree of receptor internalization defect correlated with the severity of leukopenia observed in the patients, with the E343K mutation having the least pronounced CXCR4 internalization defect associated with the mildest leukopenia phenotype. The analysis also indicated a correlation of the CXCR4 receptor internalization impairment with susceptibility to recurrent infections in patients with WHIM syndrome (Figs. 5D, S2E). CXCL12-induced AKT hyperactivation associated with lower IgA levels and decreased CD4^+^ T-cell counts (Figs. 5E, F, S2E), a finding common in activated PI3-kinase delta syndrome, which is characterized by constitutively active AKT signaling [29]. Additionally, we found a correlation between the variant’s ability to inhibit cAMP production and CD4+ T-cell counts (Fig. 5G), a finding potentially linked to the role of cAMP as an important and potent regulator of leukocyte chemotaxis [30].

## Discussion

The present study reports several novel insights in CXCR4^WHIM^ biology. The generated profiles of the 14 known pathogenic CXCR4^WHIM^ variants represent the largest dataset to date analyzing side-by side the full spectrum of CXCL12-induced events. We uncovered that, in line with previous reports [3–11], the CXCR4^WHIM^ variants share common functional characteristics consisting of impaired CXCR4 receptor internalization and degradation paralleled by enhanced signaling and chemotactic responses to CXCL12 (Figs. 2, 3). The unique parallel comparison also allowed us to identify mutation-specific trends in the magnitude and kinetics of functional responses that could be explained based on the role of various motifs in the CXCR4 C-terminus (Fig. 1A). The E343K mutation does not perturb any known functional motifs (phosphorylation, dileucine, or degradation), but can interfere with hierarchical phosphorylation events responsible for full desensitization [31]. This may underlie the milder effects on internalization, degradation, and cAMP inhibition with the E343K variant; other signaling effects induced further downstream of receptor activation (i.e., pAKT, pERK, chemotaxis) were more comparable amongst variants. E343X and S341Pfs*25 mutants retain all GRK6 phosphorylation sites in CXCR4, which may explain the partial retention of internalization at high CXCL12 concentrations. Early frameshift variants T318Pfs*3, S319Cfs*24, and V320Efs*23, lacking dileucine and degradation motifs [32], show a trend toward CXCR4 accumulation in whole-cell lysates (Fig. 2B). A decreasing E_max_ with an increasing EC_50_ was seen with these variants in Ca^2+^ mobilization assays (Fig. S1C), and T318Pfs*3 did not show ERK hyperactivation (Fig. 2F). These findings are in accordance with the reported loss of signaling in a CXCR4Δ317 mouse (corresponding to CXCR4Δ311 in humans) [33], suggesting that the region between 311 and 318 is essential for G-protein coupling and downstream signaling.

We established the first cell-based model of heterozygous c.1000C>T/R334X mutation in the endogenous locus to solve previously unaddressed questions about potential phenotypic differences between homo- and heterozygous c.1000C>T mutations. When stimulated with CXCL12, R334X/WT-expressing cells appeared to display full GOF phenotype in downstream signaling assays compared to R334X/R334X cells (Fig. 3). Such an observation is in line with the dominant character of a single mutant allele in inheritance of WHIM syndrome [34]. However, in receptor-proximal readouts (internalization, degradation), the heterozygous cells seemed to have an intermediate phenotype. Hence, partial changes in receptor activation may translate to maximal effects in receptor-distal readouts due to signal amplification [35, 36]. This observation was further supported by K562 cells expressing the E343K variant, where this mutation conferred mild effects in receptor-proximal trafficking and cAMP inhibition assays (Fig. 2D), but displayed full GOF in ERK/AKT activation upon CXCL12 stimulation (Fig. 2F).

Overall, both cellular models recapitulate well the impaired CXCR4 internalization and hyperactive receptor signaling found in immune cells from patients with WHIM syndrome [3–11]. However, cell type–specific differences were present, in particular for the calcium mobilization readout in K562 and Jurkat cells. Such differences were previously reported, showing higher calcium mobilization in patient cells harboring the R334X variant [8], even though comparable activation was detected in the Cf2Th cells expressing WT, R334X, and E343X [28]. Thus, it is possible that using different cellular models to study *CXCR4*^WHIM^ mutations may uncover more or other genotype–phenotype correlations.

We also profiled the efficacy of mavorixafor in order to confirm its activity on a wide spectrum of CXCR4^WHIM^ variants. Mutagenesis experiments have previously shown that mavorixafor interacts with residues in the ligand-biding pocket of the CXCR4 receptor [37] and thus C-terminal mutations located intracellularly were not expected to negatively impact the activity of the drug. In line with this and results from Mosi et al. [28] all CXCR4^WHIM^ mutations were sensitive to mavorixafor in downstream signaling readouts (Fig. 4). Importantly, the measured IC_50_ values for all variants in calcium mobilization assay (Fig. 4A) were below free mean plasma concentrations of mavorixafor achieved after the 400 mg once-daily dose (corrected for total bound and unbound drug) in phase 2 trial in patients with WHIM syndrome [15], suggesting the potential clinical effectiveness of mavorixafor across all known CXCR4^WHIM^ mutations. The measured potency on WT, R334X, and E343X CXCR4 in K562 cells was comparable to previously published results in Cf2Th cells and showed similar trends, i.e., lowest potency on R334X receptor and highest in WT CXCR4-expressing cells [28]. ERK and AKT activation was increased in cells expressing CXCR4^WHIM^ variants, which enabled us to analyze whether mavorixafor fully inhibits, or more preferably, normalizes CXCL12-dependent signaling to WT levels. In our studies, mavorixafor did not completely inhibit ERK/AKT activation but rather restored it to WT levels (Fig. S2C, D).

Prior to this comprehensive study of known CXCR4^WHIM^ mutations, it was not possible to directly compare phenotypes across the breadth of CXCR4 mutations found in WHIM disease. Clinical phenotypes found in patients with WHIM syndrome are highly variable and even patients harboring identical *CXCR4*^WHIM^ mutation may suffer from different disease manifestations [24]. We took a unique, quantitative approach to address the potential genotype–phenotype associations by measuring in vitro functional parameters for each CXCR4^WHIM^ variant and testing this dataset for correlations with quantifiable, clinically measured patient phenotypes available from published literature for the individual mutations (Table S5). Despite the low number of patients with certain mutations and/or incomplete patient profiles, this approach allowed us to reveal several relevant relationships (Fig. 5). Most prominent were the correlations between the magnitude of CXCR4 receptor internalization defect and the severity of neutropenia, lymphopenia, and susceptibility to recurrent infections. In addition, higher AKT activation associated with lower IgA levels and decreased CD4+ cell numbers in blood. These observations can be explained by the underlying molecular mechanisms operating downstream of CXCR4^WHIM^ mutations [5, 29] and therefore highlight the relevance of our methodology. Based on these data, we propose that CXCL12-induced CXCR4 internalization can be used as a key parameter for the assessment of *CXCR4* variant pathogenicity in vitro and as a potential disease biomarker for WHIM syndrome diagnosis. In the future, data from additional patients with WHIM syndrome having these mutations, or with milder or partial phenotypes, would be needed to further validate this approach. This work may open avenues for reclassification of variants of uncertain significance, providing a major leap forward in diagnosing and treating WHIM syndrome, WM, and other CXCR4-dependent PID diseases.

## Supplementary information

Supplementary Information

## Data Availability

Data supporting the findings of this study are available from the corresponding author on request (katarina.zmajkovicova@x4pharma.com).
