# Supplementary material for: Genotype–phenotype correlations in WHIM syndrome: a systematic characterization of CXCR4WHIM variants
Source: Genes Immun. 2022 Sep 12;23(6):196–204. doi: 10.1038/s41435-022-00181-9 (PMC9519442; doi:10.1038/s41435-022-00181-9)
Supplement: Supplementary file 1 — Supplementary Information [file 41435_2022_181_MOESM1_ESM.docx]

**Genotype–Phenotype Correlations in WHIM Syndrome: A Systematic Characterization of *CXCR4*^WHIM^ Variants: Supplementary Information Materials**

**Experimental Procedures**

***Generation of wild type (WT) and mutant CXCR4 plasmids***

The complementary DNA (cDNA) of human *CXCR4* (GenBank accession no NM_003467) carrying the consensus was synthesized by Genewiz (Leipzig, Germany) and cloned into pcDNA3.1(Hygro+) via NheI/EcoRV to give pcDNA3.1_CXCR4. The cDNA of human *CXCR4* (GenBank accession no NM_003467) carrying the consensus Kozak sequence at the 5’ end (GCCGCCACCatg) and 36 nucleotides of 3' untranslated region at the 3’ end was synthesized by Genewiz (Leipzig, Germany) and cloned into pcDNA3.1(Hygro+) via NheI/EcoRV to give pcDNA3.1_CXCR4-extended. Two of the WHIM variants, p.L329Q fs*13 and p.S339F fs*6, carrying the c.986_990delTCTCC and c.1012_1015dup mutations, respectively, were synthesized and cloned in pcDNA3.1(Hygro+), as for the WT. The S341P fs*25 variant was generated by site-directed mutagenesis using the QuickChange II XL kit (Agilent Technologies, Santa Clara, California, USA), primers listed in Table S1 and pcDNA3.1_CXCR4-extended as template. The remaining *CXCR4*^WHIM^ variants were generated by site-directed mutagenesis, using the QuickChange II XL kit (Agilent Technologies, Santa Clara, California, USA), the primers listed in Table S1, and pcDNA3.1_CXCR4 as template. Endofree Plasmid MaxiPrep Kit DNAs (Qiagen, Hilden, Germany) were generated and all plasmid preparations were confirmed by sequencing (Microsynth, Balgach, Switzerland; CMV forward, BGH reverse).

***Reagents***

Mavorixafor was synthesized by ChemPartner (Shanghai, China) and dissolved as a 10-mm stock solution in dimethyl sulfoxide (DMSO). Human CXCL12 (also known as stromal cell–derived factor 1 alpha, [SDF1α]) was purchased from PeproTech (East Windsor, New Jersey, USA).

***Cell culture and cell lines***

K562 cells (CCL-243, ATCC) were cultured in Iscove's Modified Dulbecco's Medium (IMDM [Gibco™; Thermo Fisher Scientific, Waltham, Massachusetts, USA]) supplemented with 10% fetal calf serum (Sigma-Aldrich, St Louis, Missouri, USA) and antibiotic-antimycotic (Gibco; Thermo Fisher Scientific, Waltham, Massachusetts, USA). Starvation was performed in IMDM, 0.5% bovine serum albumin (BSA; Sigma-Aldrich, St Louis, Missouri, USA), and antibiotic-antimycotic overnight to enhance the surface expression of CXCR4 and suppress serum-induced signals for subsequent signaling assays. Jurkat cells were cultured in RPMI 1640 Medium (ATCC-Modification, Gibco™; Thermo Fisher Scientific, Waltham, Massachusetts, USA), 10% FCS and penicillin/streptomycin. Starvation was performed in medium containing 0.5% BSA. Both cell lines were tested for mycoplasma contamination with negative results. Stable K562 cell lines expressing *CXCR4* were established by electroporation (Gene Pulser Xcell™ System; Bio-Rad, Hercules, California, USA) with linearized (Bgl II restriction enzyme) pcDNA3.1-CXCR4 plasmids in Ingenio^®^ Electroporation Solution (Mirus Bio, Madison, Wisconsin, USA) (Supplementary Materials). Transfected cells were selected for 14 days with 400 µg/mL hygromycin B (Invitrogen; Thermo Fisher Scientific), and then CXCR4-positive cells were sorted (BD Pharmingen™ 12G5-APC monoclonal antibody; BD Biosciences, Franklin Lakes, New Jersey, USA). Clonal cell lines with comparable CXCR4 expression were selected for further experiments (Fig. 1A). Generation of stable clones expressing the *G323Vfs*20 CXCR4* variant was unsuccessful after 2 attempts. DNA isolated from established clonal lines was sequenced (Microsynth, Balgach, Switzerland; CMV forward, BGH reverse primers) to verify the presence of correct *CXCR4* constructs in the genomic DNA. Jurkat cells (E6-1, ATCC) were generated by CRISPR-Cas9 gene editing to harbor the c.1000C>T mutation in *CXCR4* gene (resulting in R334X amino acid change) at Applied StemCell (Milpitas, California, USA) (Supplementary Materials). Single-guide RNA (ATCCTCTCCAAAGGAAAGCG) was used together with single-stranded oligodeoxynucleotide repair template for introduction of the desired mutation. Two clones were generated with homozygous mutations (clones A3, B5): 1 clone with heterozygous mutation (B2) and 1 clone edited to have silent mutations resulting in WT CXCR4 protein sequence (C3). Mutation status was verified upon cell line receipt by sequencing of the entire *CXCR4* gene.

***Calcium mobilization assay***

Stable K562 clones expressing *CXCR4* (1 × 10^5^ cells/well) were seeded in black 96-well plates with transparent bottom coated with poly-L-lysine (BioCoat^®^, Corning) and serum-starved for 24 h. Medium was removed and cells were loaded with 100 µl of fluo-4 AM (3 µM, Invitrogen) dye solution for 45 min at 37 °C. Dye solution was prepared by diluting fluo-4 powder in DMSO + 10% Pluronic F127 (Thermo Fisher Scientific, Waltham, Massachusetts, USA) to 3 mM and then preparing the final dilution in assay buffer (HBSS with Ca^2+^ and Mg^2+^, 20 mM HEPES, 0.375 g/l NaHCO_3_, 0.1% BSA + 0.77 g/L probenecid [Thermo Fisher Scientific]). Subsequently, 100 µl of assay buffer alone or assay buffer with compound dilutions was added and the plates were equilibrated in the plate reader for an additional 20 min at 37 °C. Jurkat cells (3 × 10^5^ cells/well) were incubated with the Screen Quest Calbryte-520 Probenecid-Free and Wash-Free Calcium Assay Kit (AAT Bioquest, Sunnyvale, California, USA) according to manufacturer’s instructions. CXCL12 was injected with a simultaneous measurement of fluorescent signal (FlexStation^®^ 3 Multi-Mode Microplate Reader; Molecular Devices, San Jose, California, USA). Raw traces were analyzed in SoftMax Pro 7 Software (Molecular Devices). The arbitrary units were calculated as the difference between maximal and minimal signal after treatment injection, normalized to the baseline signal before injection. Maximal effect (E_max_) and half-maximal effective and inhibitory concentrations (EC_50_ and IC_50_, respectively) were calculated in Prism (GraphPad Software, San Diego, California, USA).

***cAMP ELISA***

Stable K562 clones expressing *CXCR4* were used in ELISAs. cAMP was detected in stable K562 clones expressing *CXCR4* using cAMP-Screen™ Cyclic AMP Immunoassay System (Applied Biosystems, Thermo Fisher Scientific, Waltham, Massachusetts, USA). The clones (2 × 10^5^ cells) were starved overnight in 200 µl of starvation medium on a 96-well plate. The medium was removed and cells were first preincubated with 3-isobutyl-1-methylxanthine (0.5 mM, Sigma-Aldrich) diluted in assay buffer (HBSS with Ca^2+^ and Mg^2+^, 20 mM HEPES, 0.375 g/l NaHCO_3_, 0.1% BSA) for 15 min at 37 °C. Next, 10 µM forskolin (Sigma-Aldrich) was added either alone or together with 100 nM CXCL12 for 30 min at 37 °C. The samples were then processed according to the kit manufacturer’s instructions.

***Western blot***

Whole cell lysates were prepared by lysing stable K562 cells expressing *CXCR4* or Jurkat cells in radioimmunoprecipitation assay buffer (Sigma-Aldrich) supplemented with protease inhibitor cocktail (cOmplete; Roche CustomBiotech, Basel, Switzerland) for 30 min on ice. Lysates were centrifuged at 15,000 rpm for 10 min and the supernatants transferred to fresh tubes. After addition of sample buffer (Invitrogen 4X Bolt™ LDS Sample Buffer) and reducing agent (Invitrogen 10X Bolt™ Sample Reducing Agent), the samples were incubated at 37°C for 30 min and then run on NuPAGE 4%–12% Bis-Tris gradient gels (Invitrogen Novex™) in NuPAGE MOPS SDS running buffer. Gels were blotted on Trans-Blot^®^ Turbo™ Mini PVDF Transfer Packs (Bio-Rad) in the BioRad Turbo semidry transfer device (mixed size proteins program). Membranes were blocked in 5% nonfat milk (Sigma-Aldrich) in TBST + 0.1% Tween-20 for 15 min at room temperature and then incubated with primary antibodies overnight at 4°C. Anti-CXCR4 antibody (Product code 551852, clone 2B11, BD Biosciences) was diluted 1/750 in 5% nonfat milk in TBST + 0.1% Tween-20. Rabbit anti-rat immunoglobulin G (Product code ab6734, Abcam, Cambridge, United Kingdom) was used as a secondary antibody at 1/10,000 dilution in 5% nonfat milk in TBST + 0.1% Tween-20. Membranes were developed with enhanced chemiluminescence reagent (Amersham™ ECL Prime Western Blotting Detection Reagent; GE Healthcare, Chicago, Illinois, USA) on LAS4000 gel documentation system. Densitometry analysis of images was performed in ImageJ, and the signal from CXCL12-treated samples was compared to the untreated sample percentage of remaining CXCR4).

***Ligand binding inhibition assay***

Jurkat cells were washed once with assay buffer (HBSS + 20 mM HEPES buffer +0.2% BSA, pH 7.4) and then incubated for 15 min at room temperature with test compound diluted in assay buffer at dose-dependent concentrations. Subsequently, human CXCL12-AlexaFluor647 (26 ng/ml, Almac, Craigavon, United Kingdom) was added to the compound-preincubated cells. The cells were incubated for 30 min at room temperature. Thereafter, the cells were washed twice in assay buffer, fixed with 1% paraformaldehyde in PBS, and analyzed by flow cytometry (Cytoflex). Mean fluorescence intensity of CXCL12-AF647 was determined (FCS express software). The percentage of inhibition was calculated according to the formula: [1-((MFI-MFI_NC_)/(MFI_PC_-MFI_NC_))]*100 where MFI is the mean fluorescence intensity of cells in the presence of an inhibitor, MFI_NC_ is mean fluorescence intensity of cells in the absence of the ligand and MFI_PC_ is mean fluorescence intensity of cells in the presence of the ligand alone.

**Tables**

**Table S1** Site-directed mutagenesis primers used for *CXCR4*^WHIM^ variant generation

| Oligo Name | | DNA Sequence (5’-3’) |
| --- | --- | --- |
| R334X | F | GAT CCT CTC CAA AGG AAA GTG AGG TGG ACA TTC ATC TG |
| R334X | R | CAG ATG AAT GTC CAC CTC ACT TTC CTT TGG AGA GGA TC |
| G336X | F | CTC CAA AGG AAA GCG AGG TTG ACA TTC ATC TGT TTC CAC |
| G336X | R | GTG GAA ACA GAT GAA TGT CAA CCT CGC TTT CCT TTG GAG |
| S338X | F | GAA AGC GAG GTG GAC ATT GAT CTG TTT CCA CTG AGT C |
| S338X | R | GAC TCA GTG GAA ACA GAT CAA TGT CCA CCT CGC TTT C |
| E343X | F | GGA CAT TCA TCT GTT TCC ACT TAG TCT GAG TCT TCA AG |
| E343X | R | CTT GAA GAC TCA GAC TAA GTG GAA ACA GAT GAA TGT CC |
| E343K | F | GGA CAT TCA TCT GTT TCC ACT AAG TCT GAG TCT TCA AG |
| E343K | R | CTT GAA GAC TCA GAC TTA GTG GAA ACA GAT GAA TGT CC |
| T318P fs*3 | F | GCC CAG CAC GCA CTA CCT CTG TGA GCA G |
| T318P fs*3 | R | CTG CTC ACA GAG GTA GTG CGT GCT GGG C |
| S319C fs*24 | F | CACGCACTCACCTGTGAGCAGAGGGTC |
| S319C fs*24 | R | GACCCTCTGCTCACAGGTGAGTGCGTG |
| V320E fs*23 | F | GCA CTC ACC TCT GAG CAG AGG GTC CAG |
| V320E fs*23 | R | CTG GAC CCT CTG CTC AGA GGT GAG TGC |
| G323V fs*20 | F | CCT CTG TGA GCA GGG TCC AGC CTC AAG |
| G323V fs*20 | R | CTT GAG GCT GGA CCC TGC TCA CAG AGG |
| S324V fs*20 | F | CTG TGA GCA GAG GGG TCC AGC CTC AAG ATC |
| S324V fs*20 | R | GAT CTT GAG GCT GGA CCC CTC TGC TCA CAG |
| S339C fs*4 | F | CGA GGT GGA CAT TCA TGT TTC CAC TGA GTC TG |
| S339C fs*4 | R | CAG ACT CAG TGG AAA CAT GAA TGT CCA CCT CG |
| S341P fs*25 | F | GTG GAC ATT CAT CTG TTC CAC TGA GTC TGA GTC |
| S341P fs*25 | R | GAC TCA GAC TCA GTG GAA CAG ATG AAT GTC CAC |

WHIM, Warts, Hypogammaglobulinemia, Infections, and Myelokathexis.

**Table S2** An overview of *in vitro* variant characterization assay results

|  | | **Internalization** | | **Degradation** | | **cAMP** | **ERK** | | | **AKT** | | **Chemotaxis** | **Ca flux** | |
| --- | --- | --- | --- | --- | --- | --- | --- | --- | --- | --- | --- | --- | --- | --- |
| **Variants** | |  | |  | | **Inhibition** | **Amplitude** | **Duration** | | **Amplitude** | **Duration** |  | **E_max_ (%)** | **EC_50_ (nM)** |
| WT | |  | |  | |  |  |  | |  |  |  | 100 | 1.59 |
| R334X | | ↓↓ | | ↓ | | ↑ | ↑ | ↑ | | = | ↑ | ↑ | 117 | 1.63 |
| G336X | | ↓↓ | | ↓ | | ↑ | ↑ | = | | ↑ | ↑ | ↑↑ | 102 | 1.72 |
| S338X | | ↓↓ | | ↓ | | ↑ | ↑ | ↑ | | ↑ | ↑ | ↑ | 68 | 0.73 |
| E343X | | ↓↓ | | ↓ | | ↑ | ↑ | ↑ | | ↑ | ↑ | ↑ | 86 | 1 |
| E343K | | ↓ | | = | | ↑ | ↑ | ↑ | | ↑ | ↑ | ↑ | 95 | 1.16 |
| T318P fs*3 | | ↓↓ | | ↓ | | ↑ | = | = | | ↑ | ↑ | ↑ | 73 | 5.1 |
| S319C fs*24 | | ↓↓ | | ↓ | | ↑ | ↑ | ↑ | | ↑ | ↑ | ↑ | 83 | 2.96 |
| V320E fs*23 | | ↓↓ | | ↓ | | ↑ | ↑ | ↑ | | ↑ | ↑ | ↑ | 105 | 2.27 |
| G323V fs*20 | | ↓↓ | |  | |  |  |  | |  |  | ↑↑ |  |  |
| S324V fs*20 | | ↓↓ | | ↓ | | ↑ | ↑ | = | | ↑ | ↑ | ↑↑ | 110 | 0.61 |
| L329Q fs*13 | | ↓↓ | | ↓ | | ↑ | ↑ | ↑ | | ↑ | ↑ | ↑↑ | 78 | 0.54 |
| S339C fs*4 | | ↓↓ | | ↓ | | ↑ | ↑ | = | | ↑ | = | ↑↑ | 92 | 0.78 |
| S339F fs*6 | | ↓↓ | | ↓ | | ↑ | ↑ | ↑ | | ↑ | ↑ | ↑↑ | 101 | 0.9 |
| S341P fs*25 | | ↓↓ | | ↓ | | ↑ | = | ↑ | | ↑ | ↑ | ↑↑ | 104 | 4.66 |
|  |  | |  | |  | | | |  |  |  |  |  |  |
| legend | ↑/↓ | | Increased/decreased in 1/few conditions | | | | | |  |  |  |  |  |  |
|  | ↓↓/↑↑ | | Increased/decreased in all conditions | | | | | |  |  |  |  |  |  |
|  | = | | Comparable to WT | | | | | |  |  |  |  |  |  |
|  |  | |  | |  | | | |  |  |  |  |  |  |

AKT, protein kinase B; cAMP, cyclic adenosine monophosphate; EC_50,_ half-maximal effective concentration; E_max_, maximum effect; ERK, extracellular signal-regulated kinase; WT, wild-type.

**Table S3** An overview of *in vitro* characterization of Jurkat R334X cell lines

|  | Ca^2+^ mobilization (CXCL12) | | CXCL12 binding inhibition (mavorixafor) | Ca^2+^ mobilization (mavorixafor) | Chemotaxis (mavorixafor) |
| --- | --- | --- | --- | --- | --- |
| Cell line | **EC_50_  (nM ± SE)** | **E_max_  (% ± SE)** | **IC_50_  (nM ± SE)** | **IC_50_  (nM ± SE)** | **IC_50_  (nM ± SE)** |
| **Parental** | 5.6 ± 2.7 | 98 ± 7.5 | 1.9 ± 0.09 | 1.5 ± 0.9 | - |
| **WT/WT (C3)** | 8.1 ± 3.2 | 117 ± 9.2 | 1.6 ± 0.12 | 0.7 ± 0.3 | 30 ± 10 |
| **RX/WT (B2)** | 13.8 ± 9.5 | 273 ± 40 | 2.4 ± 0.07 | 3.4 ± 3.6 | 366 ± 334 |
| **RX/RX (A3)** | 7.6 ± 4.5 | 310 ± 34 | 2.1 ± 0.1 | 5 ± 2.8 | 188 ± 20 |
| **RX/RX (B5)** | 5.1 ± 3.1 | 283 ± 28 | 1.9 ± 0.15 | 2 ± 1.2 | 153 ± 23 |

CXCL12, C-X-C chemokine ligand 12; EC_50_, half-maximal effective concentration; E_max_, maximum effect; IC_50,_ half-maximal inhibitory concentration; SEM, standard error of mean; WT, wild-type; RX, R334X.

**Table S4** Hematology and serology measurements and clinical phenotypes (warts, hypogammaglobulinemia, recurrent infections, myelokathexis, expressed as fraction of patients) reported in patients carrying *CXCR4*^WHIM^ mutations

|  |  |  | Cells/ul | Cells/ul | Cells/ul | Cells/ul | Cells/ul | Cells/ul | Cells/ul | Cells/ul | mg/dl | mg/dl | mg/dl | Cells/ul |
| --- | --- | --- | --- | --- | --- | --- | --- | --- | --- | --- | --- | --- | --- | --- |
| **Patient** | **Mutation** | **Reference** | **WBC** | **ANC** | **AMC** | **ALC** | **CD3** | **CD4** | **CD8** | **CD19** | **IgG** | **IgA** | **IgM** | **Platelets** |
| 1 | S319C fs*24 | Dr. Sharathkumar (personal) | 5000 | 89 | 536 | 1100 | 935 | 44 | 88 | 44 | 959 | 5 | 37 | 116 |
| 2 | S319C fs*24 | Moens, *J Allergy Clin Immunol*. 2016;138:1485 |  | 590 | 300 | 600 | 424 | 308 | 89 | 25 |  |  |  | 308 |
| **2** | S319C fs*24 | **AV** | **5000** | **340** | **418** | **850** | **680** | **176** | **89** | **35** | **959** | **5** | **37** | **212** |
|  |  | **SD** |  | **354** | **167** | **354** | **361** | **187** | **1** | **13** |  |  |  | **136** |
|  |  |  |  |  |  |  |  |  |  |  |  |  |  |  |
| 1 | V320E fs*23 | Gernez *J Clin Immunol*.2019;39(Suppl 1):1 | 3500 | 700 | 500 | 1058 | 899 | 423 |  | 11 | 518 | 62 | 108 | 5 |
| 2 | V320E fs*23 | Gernez *J Clin Immunol*.2019;39(Suppl 1):1 | 2200 | 550 | 176 | 285 | 248 | 148 |  | 11 | 755 | 133 | 210 | 189 |
| **2** | V320E fs*23 | AV | **2850** | **625** | **338** | **672** | **574** | **286** |  | **11** | **637** | **98** | **159** | **97** |
|  |  | SD | **919** | **106** | **229** | **547** | **460** | **194** |  |  | **168** | **50** | **72** | **130** |
|  |  |  |  |  |  |  |  |  |  |  |  |  |  |  |
| 1 | G323V fs*20 | Beaussant Cohen, *Orphanet J Rare Dis.* 2012;7 |  | 160 | 80 | 380 | 330 | 200 | 100 |  | 570 | 53 | 55 | 223 |
| 2 | G323V fs*20 | Bhar, *Blood.* 2015;136:5528 | 1003 | 70 |  |  |  |  |  |  |  |  |  | 70 |
| 3 | G323V fs*20 | Shin, *Ann Lab Med.* 2017;37:446 |  |  |  |  |  |  |  |  | 282 | 19 | 59 |  |
| **3** | G323V fs*20 | AV | **1003** | **115** | **80** | **380** | **330** | **200** | **100** |  | **426** | **36** | **57** | **147** |
|  |  | SD |  | **64** |  |  |  |  |  |  | **204** | **24** | **3** | **108** |
|  |  |  |  |  |  |  |  |  |  |  |  |  |  |  |
| **1** | S324V fs*20 | McDermott, *NEJM.* 2019;380:163 | **3000** | **50** | **300** | **1320** | **600** | **250** | **350** | **300** | **1100** | **100** | **80** | **200** |
|  |  |  |  |  |  |  |  |  |  |  |  |  |  |  |
| **1** | L329Q fs*13 | Liu, *JCI.* 2016;36:397 | **900** | **140** | **40** | **700** | **572** | **426** | **92** | **13** | **normal** |  |  |  |
|  |  |  |  |  |  |  |  |  |  |  |  |  |  |  |
| 1 | R334x | Dotta, *JACI.* 2019;7:1568 Gulino, *Blood.* 2004;104:444 | 1510 | 300 | 60 | 1009 | reduced |  |  | reduced | 377 | 5 | 44 |  |
| 2 | R334x | Dotta, *JACI*. 2019;7:1568 | 800 | 76 | 33 | 596 |  |  |  |  | 579 | 135 | 59 |  |
| 3 | R334x | Dotta, *JACI.* 2019;7:1568 |  |  |  |  |  |  |  |  |  |  |  |  |
| 4 | R334x | Dotta, *JACI.* 2019;7:1568 | 1120 | 232 | 139 | 726 |  |  |  |  | 790 | 130 | 160 |  |
| 5 | R334x | Dotta, *JACI.* 2019;7:1568 | 790 | 90 | 70 | 600 |  |  |  |  | 415 | 11 | 25 |  |
| 6 | R334x | Dotta, *JACI.* 2019;7:1568 | 1700 | 300 | 100 | 1275 |  |  |  |  | 413 | 48 | 54 |  |
| 7 | R334x | Dotta, *JACI.* 2019;7:1568 | 2200 | 311 | - | 1636 |  |  |  |  | 178 | 11 | 119 |  |
| 8 | R334x | Siedlar, *Arch Immunol Ther Exp.* 2008;56:419 |  | 172 |  | 662 | 171 | 57 | 93 |  | 430 | 37 | 38 |  |
| 9 | R334x | Beaussant Cohen, *Orphanet J Rare Dis.* 2012;7 |  | 160 | 100 | 1270 | 1000 | 770 | 130 | 70 | 283 | 0 | 53 | 367 |
| 10 | R334x | Taniuchi, *Am J Hematol*. 1999;62:106 | 2000 | 480 | 260 | 1260 | 58 | 45 | 15 |  | 405 | 21 | 191 | 186 |
| 11 | R334x | Taniuchi, *Am J Hematol.* 1999;62:106 | 1400 | 60 | 40 | 1220 | 68 | 52 | 17 |  | 458 | 29 | 190 | 642 |
| 12 | R334x | Gorlin, *Am J Med Gen.* 2000;91:368 | 3400 | 646 | 442 | 2176 | 416 |  |  | 20 | 826 | 224 | 30 |  |
| 13 | R334x | Wetzler, *Am J Med*. 1990;89:663 | 1000 | 270 | 70 | 650 | 470 | 222 | 250 | 9 | 568 | 62 | 28 | 395 |
| 14 | R334x | Wetzler, *Am J Med*. 1990;89:663 | 1000 | 130 | 110 | 750 | 480 | 326 | 191 | 16 | 386 | 56 | 39 | 248 |
| 15 | R334x | Chen, *PMID*: 23751577 | 650 | 150 |  |  | 218 |  |  | 5 | 556 | 48 | 29 | 200 |
| 16 | R334x | Bock, *Ann Biol Clin*. 2014;72:111 | 1000 | 240 |  | 520 | 360 | 260 | 80 | 10 |  |  |  |  |
| 17 | R334x | McDermott, *Rare Dis*. 2015;e1073430 |  | 380 |  |  | 382 | 317 | 41 | 5 |  |  |  |  |
| 18 | R334x | McDermott, *Rare Dis.* 2015;e1073430 |  | 530 |  |  | 218 | 155 | 45 | 10 |  |  |  |  |
| 19 | R334x | Aghamohammadi, *JCI.* 2017;37:282 | 2080 | 790 |  | 1630 | 1402 | 408 | 848 | 130 | 386 | 10 | 57 |  |
| 20 | R334x | Kawahara, *JCI*. 2018; doi/10.1007/s10875-018-0529-4 | 2000 | 0 | 40 | 1920 |  |  |  |  | 270 | 48 | 61 | 247 |
| **20** | **R334x** | **AV** | **1510** | **280** | **122** | **1119** | **437** | **261** | **171** | **31** | **458** | **55** | **74** | **326** |
|  |  | **SD** | **737** | **208** | **118** | **516** | **394** | **219** | **250** | **43** | **173** | **60** | **58** | **160** |
|  |  |  |  |  |  |  |  |  |  |  |  |  |  |  |
| **1** | **G336x** | Dotta, *JACI.* 2019;7:1568 Gulino, *Blood.* 2004;104:444 | **940** | **150** | **34** | **700** | **reduced** |  |  | **reduced** | **810** | **99** | **26** |  |
|  |  |  |  |  |  |  |  |  |  |  |  |  |  |  |
| 1 | S338x | Dotta, *JACI.* 2019;7:1568 | 800 | 208 | - | 401 |  |  |  |  | 1137 | 243 | 256 |  |
| 2 | S338x | Dotta, *JACI.* 2019;7:1568 | - | - | - | - |  |  |  |  | 955 | 288 | 86 |  |
| 3 | S338x | Dotta, *JACI.* 2019;7:1568 | 650 | 256 | 24 | 320 |  |  |  |  | 807 | 83 | 238 |  |
| 4 | S338x | Dotta, *JACI.* 2019;7:1568 | 760 | 60 | 100 | 440 |  |  |  |  | 557 | 72 | 129 |  |
| 5 | S338x | Dotta, *JACI.* 2019;7:1568 | 1100 | 132 | 132 | 682 |  |  |  |  | 355 | 25 | 67 |  |
| 6 | R338x | Dotta, *JACI.* 2019;7:1568 | - | 300 | - | 1250 |  |  |  |  | 843 | 125 | 175 |  |
| 7 | S338x | Beaussant Cohen, *Orphanet J Rare Dis.* 2012;7 |  | 220 | 90 | 580 | 970 | 300 | 420 | 10 | 970 | 189 | 25 | 202 |
| 8 | S338x | Beaussant Cohen, *Orphanet J Rare Dis.* 2012;7 |  | 400 | 70 | 170 | 300 | 40 | 40 | 0 | 420 | 50 | 40 | 212 |
| 9 | S338x | Beaussant Cohen, *Orphanet J Rare Dis.* 2012;7 |  | 340 | 120 | 420 | 330 | 150 | 190 | 40 | 720 | 0 | 0 | 193 |
| 10 | S338x | Beaussant Cohen, *Orphanet J Rare Dis.* 2012;7 |  | 140 | 100 | 1200 | 600 | 540 | 40 | 0 | 180 | 100 | 0 | 306 |
| 11 | S338x | Alapi, *Eur J Hematol*. 2007;78:86 |  |  |  | 1150 | 932 | 506 | 138 | 26 |  |  |  |  |
| **11** | **S338x** | **AV** | **828** | **228** | **91** | **661** | **626** | **307** | **166** | **15** | **694** | **118** | **102** | **228** |
|  |  | **SD** | **192** | **108** | **36** | **397** | **319** | **218** | **156** | **18** | **307** | **95** | **94** | **52** |
|  |  |  |  |  |  |  |  |  |  |  |  |  |  |  |
| 1 | S339C fs*4 | Dotta, *JACI.* 2019;7:1568 | 510 | 150 | 40 | 300 |  |  |  |  | 967 | 75 | 161 |  |
| 2 | S339C fs*4 | Dotta, *JACI.* 2019;7:1568 | 1500 | 300 | 100 | 1000 |  |  |  |  | - | - | - |  |
| **2** | S339C fs*4 | **AV** | **1005** | **225** | **70** | **650** |  |  |  |  | **967** | **75** | **161** |  |
|  |  | **SD** | **700** | **106** | **42** | **495** |  |  |  |  |  |  |  |  |
|  |  |  |  |  |  |  |  |  |  |  |  |  |  |  |
| **1** | S341P fs*25 | Dotta, *JACI.* 2019;7:1568 | **2900** | **377** | **-** | **2320** |  |  |  |  | **375** | **42** | **87** |  |
|  |  |  |  |  |  |  |  |  |  |  |  |  |  |  |
| 1 | E343x | Hord, *J Pediatr Hematol Oncol.* 1997;19:43 | 600 | 750 |  |  | normal |  |  | normal | 670 | 0 | normal |  |
| 2 | E343x | Hord, *J Pediatr Hematol Oncol*, 1997;19:43 |  | 600 |  |  |  |  |  |  | 497 |  |  |  |
| **2** | **E343x** | **AV** | **600** | **675** |  |  |  |  |  |  | **584** |  |  |  |
|  |  | **SD** |  | **106** |  |  |  |  |  |  | **122** |  |  |  |
|  |  |  |  |  |  |  |  |  |  |  |  |  |  |  |
| 1 | E343K | Liu, *Blood.* 2012;120:181 | 2570 | 1060 | 200 | 1230 | 943 | 478 | 347 | 127 | 694 | 170 | 70 |  |
| 2 | E343K | Liu, *Blood.* 2012;120:181 | 3390 | 1050 | 270 | 1980 | 1497 | 903 | 449 | 414 | 791 | 95 | 72 |  |
| 3 | E343K | Liu, *Blood.* 2012;120:181 | 3070 | 1950 | 160 | 910 | 700 | 512 | 161 | 152 | 705 | 103 | 113 |  |
| 4 | E343K | Liu, *Blood.* 2012;120:181 | 2930 | 1450 | 240 | 1190 |  |  |  |  | 725 | 103 | 71 |  |
| **4** | **E343K** | **AV** | **2990** | **1378** | **218** | **1328** | **1047** | **631** | **319** | **231** | **729** | **118** | **83** |  |
|  |  | **SD** | **340** | **425** | **48** | **458** | **409** | **236** | **146** | **159** | **43** | **35** | **21** |  |
|  |  |  |  |  |  |  |  |  |  |  |  |  |  |  |
|  |  | normal level adult | 4500–11,000 | 2500–6000 | 700–900 | 1000–4000 | 655–2823 | 321–1389 | 220–1664 | 103–581 | 600–1600 | 80–300 | 60–250 |  |
|  |  | AV | **7750** | **4250** | **800** | **2500** | **1739** | **855** | **942** | **342** | **1100** | **190** | **155** |  |
|  |  | SD | **1658** | **892** | **51** | **765** | **553** | **272** | **368** | **121** | **255** | **56** | **48** |  |

| Variants | **Warts**  **(Fraction of Patients)** | **Hypogammaglobulinemia**  **(Fraction of Patients)** | **Infection**  **(Fraction of Patients)** | **Myelokathexis**  **(Fraction of Patients)** | **Total Number of Patients** |
| --- | --- | --- | --- | --- | --- |
| WT |  |  |  |  |  |
| R334X | 0.68 | 0.81 | 0.87 | 0.98 | 47 |
| G336X | 1.00 | 1.00 | 1.00 | 1.00 | 2 |
| S338X | 0.33 | 0.53 | 0.87 | 1.00 | 15 |
| E343X | 0.50 | 1.00 | 1.00 | 1.00 | 2 |
| E343K | 0.25 | 0.25 | 0.75 | 1.00 | 4 |
| T318P fs*3 (1) |  |  |  |  | 3 |
| S319C fs*24 (2) | 1.00 | 1.00 | 1.00 | 1.00 | 1 |
| V320E fs*23 (3) | 0.00 | 1.00 | 1.00 |  | 2 |
| G323V fs*20 | 0.40 | 0.60 | 0.60 | 0.80 | 5 |
| S324V fs*20 | 0.00 | 1.00 | 1.00 | 1.00 | 1 |
| L329Q fs*13 | 1.00 | 1.00 | 1.00 | 1.00 | 1 |
| S339C fs*4 | 0.67 | 0.67 | 1.00 | 1.00 | 6 |
| S339F fs*6 (4) | 1.00 | 0.00 | 1.00 | 1.00 | 1 |
| S341P fs*25 | 1.00 | 0.00 | 1.00 | 1.00 | 1 |

ALC, absolute lymphocyte count; AMC, absolute monocyte count; ANC, absolute neutrophil count; AV, average value; CD, cluster of differentiation; IgG, immunoglobulin G; IgM, immunoglobulin M; WBC, white blood cell; WHIM, Warts, Hypogammaglobulinemia, Infections, and Myelokathexis; WT, wild-type.

Data from Heusinkveld LE, Majumdar S, Gao J-L, McDermott DH. WHIM syndrome: from pathogenesis towards personalized medicine and cure. *J Clin Immunol*. 2019;39(6):532-556, except for:

1. Dotta L, Notarangelo LD, Porta F, Soresina A, Lougaris V, Plebani A, et al. WHIM syndrome: clinical phenotype and therapeutic measures of a cohort of 21 patients. Presented at the 17th Biennial Meeting of the European Society for Immunodeficiencies (ESID); September 21–24, 2016; Barcelona, Spain.
2. Moens L, Frans G, Bosch B, Bossuyt X, Verbinnen B, Poppe W, et al. Successful hematopoietic stem cell transplantation for myelofibrosis in an adult with warts-hypogammaglobulinemia-immunodeficiency-myelokathexis syndrome [abstract]. *J Allergy Clin Immunol*. 2016;138(5):1485-1489.
3. Gernez Y, Chavez J, Bussel J, Cunningham-Rundles C. Unexpected diagnosis in a family with autoimmune multilineage cytopenia and hypogammaglobulinemia. *J Clin Immunol*. 2019;39(1 suppl):1-2.
4. Luo J, de Pascali F, Richmond GW, Khojah AM, Benovic JL. Characterization of a new WHIM syndrome mutant reveals mechanistic differences in regulation of the chemokine receptor CXCR4. *J Biol Chem.* 2022;298(2):101551.

**Table S5** Significance level, expressed as *P* values, of pairwise correlations [Pearson (A, B) and Spearman (C,D)] between clinical and *in vitro* functional data for the *CXCR4*^WHIM^ mutations. Significance level, expressed as *P* values, of pairwise correlations (Pearson) between parameters measured *in vitro* (see table legend for details).

1. **Pearson correlations (excluding WT)**

|  | ERK 5 min | AKT 5 min | cAMP | ERK 30 min | AKT 30 min | Degradation | Chemotaxis 0.4 nM | Chemotaxis 2 nM | Chemotaxis 10 nM | Internalization 45 min | Internalization 4h | Internalization 45 min 11 nM CXCL12 | Internalization 4h 11 nM CXCL12 |
| --- | --- | --- | --- | --- | --- | --- | --- | --- | --- | --- | --- | --- | --- |
| WBC | ns | ns | ns | ns | ns (0.0985) | ns | ns | ns | ns | ns | ns | ns | ns |
| ANC | ns | ns | ns (0.0567) | ns | ns | ns | ns | ns | ns | **** | *** | **** | * |
| AMC | ns | ns | ns | ns | ns | ns (0.0694) | ns | ns | ns | ns | ns | ns | ns |
| ALC | ns | ns | ns | ns | ns | ns | ns | ns (0.0842) | ns | ns | ns | ns | ns |
| CD3 | ns | ns | ns (0.0592) | ns | ns | ns | ns | ns | ns | * | * | ** | ns |
| CD4 | ns | ns | *** | ns | ns (0.0504) | ns | ns | ns | ns | ** | * | ** | * |
| CD8 | ns | ns | ns | ns | ns | ns | ns | ns | ns | ns | ns | ns | ns |
| CD19 | ns | ns | ns | ns | ns | ns | * | ns | ns | ns | ns | ns | ns |
| IgG | ns | ns | ns | ns | ns | ns | ns | ns | ns | ns | ns | ns | ns |
| IgA | ns | ns | ns | ns | ** | ns | ns | ns | ns | ns | ns | ns | ns |
| IgM | ns | ns | ns | ns | ns | ns | ns | ns | ns | ns | ns | ns | ns |
| Warts | ns (0.0706) | ns | ns | ns | ns | ns | ns | ns | ns | ns | ns | ns | ns |
| Hypogamm | ns | ns | ns | ns | ns | ns | * | * | ns | ns | ns | ns | ns |
| Infections | ns | ns | ns | ns | ns | * | ns | ns | * | ** | ** | ** | * |

1. **Pearson correlations (including WT)**

|  | ERK 5 min | AKT 5 min | cAMP | ERK 30 min | AKT 30 min | Degradation | Chemotaxis 0.4 nM | Chemotaxis 2 nM | Chemotaxis 10 nM | Internalization 45 min | Internalization 4h | Internalization 45 min 11 nM CXCL12 | Internalization 4h 11 nM CXCL12 |
| --- | --- | --- | --- | --- | --- | --- | --- | --- | --- | --- | --- | --- | --- |
| WBC | ns | ns | ns | ns | ns | ns | ns | ns | ns | ns | ns | ns | ** |
| ANC | ns | ns | ** | ns | ns | * | ns | ns | ns | *** | *** | *** | **** |
| AMC | ns | ns | ns | ns | ns | ns | ns | * | ns | ns | ns | ns | ** |
| ALC | * | ns | ns | ns | ns | ns | ns | ns | ns | ns | ns | ns | ns |
| CD3 | ns | ns | ** | ns | ns | ns | ns | ns | ns | ** | ** | ** | *** |
| CD4 | ns | ns | **** | ns | * | * | ns | ns | ns | ** | *** | *** | *** |
| CD8 | * | ns | ns | ns | ns | * | ns | ns | ns | ns | ns | * | ** |
| CD19 | ns | ns | ns | * | ns | ns | ns | ns | ns | ns | ns | * | ns |
| IgG | ns | ns | ns | ns | ns | ns | ns | ns | ns | ns | ns | ns | ns |
| IgA | ns | ns | * | ns | ** | ns | ns | ns | ns | * | * | * | * |
| IgM | ns | ns | ns | ns | ns | ns | ns | ns | ns | ns | ns | ns | ns |
| Warts | ns | ns | ns | ns | ns | ns | ns | ns | ns | ns | ns | ns | ns |
| Hypogamm | ns | ns | ns | ns | ns | ns | ns | ns | ns | ns | ns | ns | ns |
| Infections | ns | * | ** | ns | ns | ** | ns | ns | * | ** | *** | *** | **** |

1. **Spearman correlations (excluding WT)**

|  | ERK 5 min | AKT 5 min | cAMP | ERK 30 min | AKT 30 min | Degradation | Chemotaxis 0.4 nM | Chemotaxis 2 nM | Chemotaxis 10 nM | Internalization 45 min | Internalization 4h | Internalization 45 min 11 nM CXCL12 | Internalization 4h 11 nM CXCL12 |
| --- | --- | --- | --- | --- | --- | --- | --- | --- | --- | --- | --- | --- | --- |
| WBC | ns | ns | ns | ns | ns | ns | ns | ns | ns | ns | ns | ns | ns |
| ANC | ns | ns | ns | ns | ns | ns | ns | ns | ns | ns | * | ns | ns |
| AMC | ns | ns | ns | ns | ns | ns | ns | ns | ns | ns | ns | ns | ns |
| ALC | ns | ns | ns | ns | ns | ns | ns | ns | ns | ns | ns | ns | ns |
| CD3 | ns | ns | ns | ns | ns | ns | ns | ns | ns | ns | ns | ns | ns |
| CD4 | ns | ns | ** | ns | ** | ns | ns | ns | ns | ns | ns | ns | ns |
| CD8 | ns | ns | ns | ns | ns | ns | ns | ns | ns | ns | ns | ns | ns |
| CD19 | ns | ns | ns | ns | ns | ns | ns | ns | ns | ns | ns | ns | ns |
| IgG | ns | ns | ns | ns | ns | ns | ns | ns | ns | ns | ns | ns | ns |
| IgA | ns | ns | ns | ns | ns | ns | ns | ns | ns | ns | ns | ns | ns |
| IgM | ns | ns | ns | ns | ns | ns | ns | ns | ns | ns | ns | ns | ns |
| Warts | ns | ns | ns | ns | ns | ns | ns | ns | ns | ns | ns | ns | ns |
| Hypogamm | ns | ns | ns | ns | ns | ns | ns | ns | ns | ns | ns | ns | ns |
| Infections | ns | ns | ns | ns | ns | * | ns | ns | * | ns | ns | ns | ns |

1. **Spearman correlations (including WT)**

|  | ERK 5 min | | AKT 5 min | cAMP | ERK 30 min | AKT 30 min | Degradation | Chemotaxis 0.4 nM | Chemotaxis 2 nM | Chemotaxis 10 nM | Internalization 45 min | Internalization 4h | Internalization 45 min 11 nM CXCL12 | Internalization 4h 11 nM CXCL12 |
| --- | --- | --- | --- | --- | --- | --- | --- | --- | --- | --- | --- | --- | --- | --- |
| WBC | | ns | ns | ns | ns | ns | ns | ns | ns | ns | ns | ns | ns | ns |
| ANC | | ns | ns | ns | ns | ns | ns | ns | ns | ns | * | * | * | ns |
| AMC | | ns | ns | ns | ns | ns | ns | ns | * | ns | ns | ns | ns | ns |
| ALC | | ns | ns | ns | ns | ns | ns | ns | ns | ns | ns | ns | ns | ns |
| CD3 | | ns | ns | ns | ns | ns | ns | ns | ns | ns | ns | ns | ns | ns |
| CD4 | | ns | ns | ** | ns | *** | ns | ns | ns | ns | * | * | ns | * |
| CD8 | | ns | ns | ns | ns | ns | * | ns | ns | ns | ns | ns | ns | ns |
| CD19 | | ns | ns | ns | ns | ns | ns | ns | ns | ns | ns | ns | ns | ns |
| IgG | | ns | ns | ns | ns | ns | ns | ns | ns | ns | ns | ns | ns | ns |
| IgA | | ns | ns | ns | ns | ** | ns | ns | ns | ns | ns | ns | ns | ns |
| IgM | | ns | ns | ns | ns | ns | ns | ns | ns | ns | ns | ns | ns | ns |
| Warts | | ns | ns | ns | ns | ns | ns | ns | ns | ns | ns | ns | ns | ns |
| Hypogamm | | ns | ns | ns | ns | ns | ns | ns | ns | ns | ns | ns | ns | ns |
| Infections | | ns | ns | ns | ns | ns | ** | ns | ns | ** | * | * | ns | ** |
|  | |  |  |  |  |  |  |  |  |  |  |  |  |  |

| **Legend** |  | Highly significant in correlations both including and excluding *CXCR4*^WT^, shown in the main manuscript |
| --- | --- | --- |
|  |  | Significant in correlations both including and excluding *CXCR4*^WT^, not shown in the main manuscript |
|  |  | Shown in manuscript |

ALC, absolute lymphocyte count; ANC, absolute neutrophil count; AMC, absolute monocyte count; CD, cluster of differentiation; ERK, extracellular signal regulated kinase; IgA, immunoglobulin A; IgG, immunoglobulin G; IgM, immunoglobulin M; ns, not significant; WBC, white blood cell; WT, wild-type.

**Supplementary Figures**

**Fig. S1A** CXCR4 surface expression in stable clonal K562 lines expressing the indicated CXCR4 constructs. Surface expression was measured by flow cytometry and is shown as MFI of CXCR4 staining. Each bar represents individual clonal line. Mean +/- SEM, n=5–16. **B** Transiently transfected K562 cells were stimulated with CXCL12 (vehicle, 1 nM, 10 nM, 100 nM) for 45 min or 4 h, and the surface expression of CXCR4 was measured by flow cytometry. Values are expressed as % remaining CXCR4 compared to vehicle-treated cells. Values represent mean +/- SEM of 4 independent experiments. **C** E_max_ (% WT) and EC_50_ values of CXCL12 determined in Ca^2+^ mobilization assays. K562 cells with stable CXCR4 expression were stimulated with serial dilutions of CXCL12. Mean +/- SEM, n=14–57. CXCL12, C-X-C chemokine ligand 12; CXCR4, C-X-C chemokine receptor 4; EC_50_, half-maximal effective concentration; E_max_, maximum effect; FC, fold change; MFI, mean fluorescence intensity; SEM, standard error of mean; WT, wild-type.

**
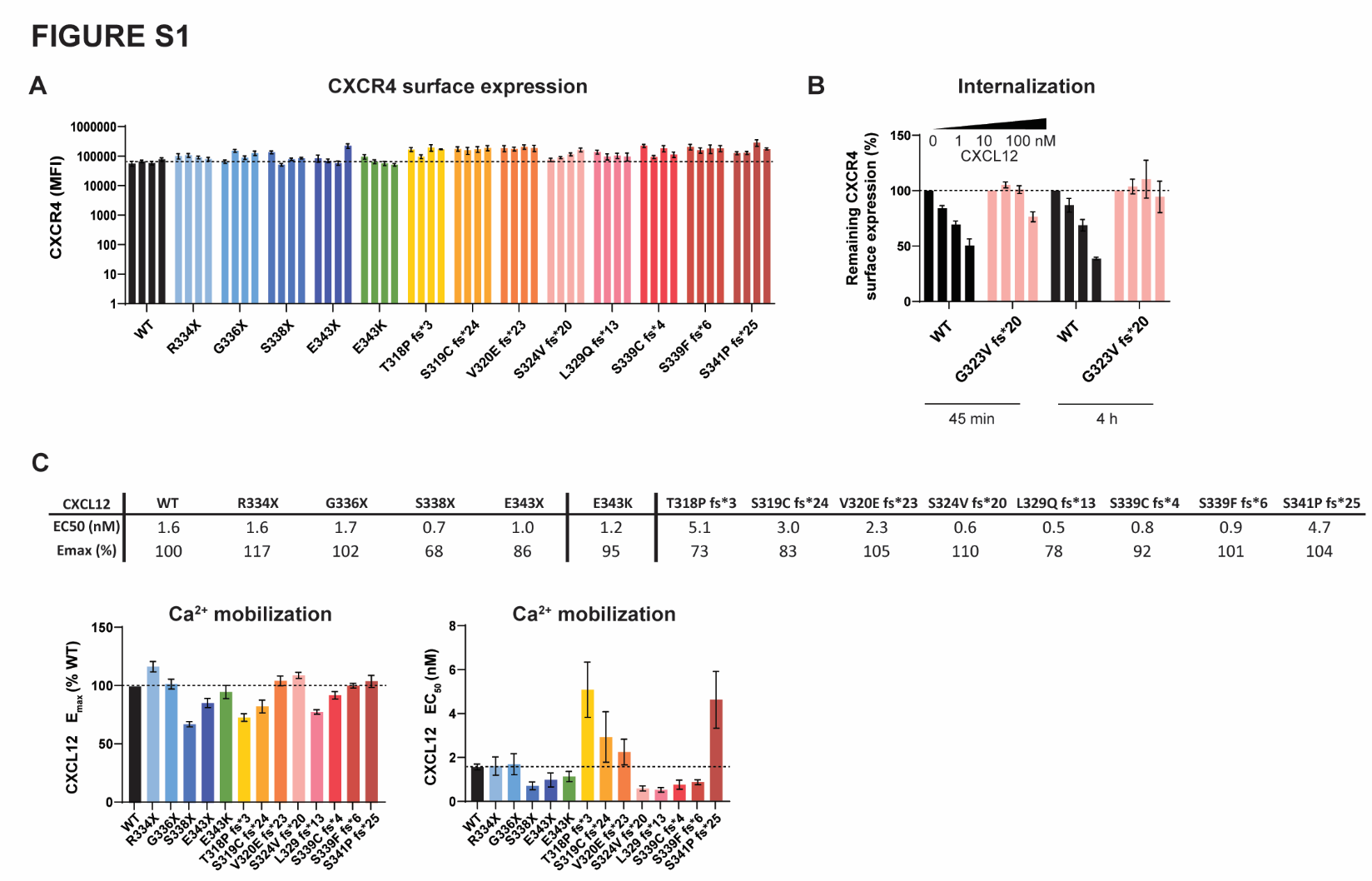
**

**Fig. S2A** E_max_ (% inhibition) and IC_50_ values of mavorixafor determined in Ca^2+^ mobilization assays. K562 cells with stable CXCR4 expression were preincubated with serial dilutions of mavorixafor and then stimulated with 10 nM CXCL12. Mean +/- SEM, n=4–17. **B** % inhibition of Ca^2+^ mobilization induced by CXCL12. K562 cells with stable CXCR4 expression were preincubated with 150 nM mavorixafor and then stimulated with 1 nM or 100 nM CXCL12. Mean +/- SEM, n=9–54. **C** Profiling of mavorixafor in ERK activation assay. K562 cells with stable CXCR4 expression were preincubated with 40 nM, 200 nM, or 1 µM mavorixafor and then stimulated with 10 nM CXCL12 for 5 min. The MFI of p-T202/Y204 ERK staining was measured by flow cytometry. Values are expressed as FC from baseline (vehicle-treated cells, no CXCL12). Mean +/- SEM, n=3–17. **D** Profiling of mavorixafor in AKT activation assay. K562 cells with stable CXCR4 expression were preincubated with 40 nM, 200 nM, or 1 µM mavorixafor and then stimulated with 10nM CXCL12 for 5 min. MFI of p-S473 AKT staining was measured by flow cytometry. Values are expressed as FC from baseline (vehicle-treated cells, no CXCL12). Mean +/- SEM, n=3–14. **E** Plots showing correlation between ANC levels/CXCR4 internalization, CD4^+^ cells/CXCR4 internalization, CD3^+^ cells/CXCR4 internalization, recurrent infections/CXCR4 internalization, and CXCR4 degradation/recurrent infections. Linear regression was used to analyze the correlation of measured values. Values are plotted as mean +/-SD. AKT, protein kinase B; ANC, absolute neutrophil count; CD, cluster of differentiation; CXCL12, C-X-C chemokine ligand 12; CXCR4, C-X-C chemokine receptor 4; EC_50_, half-maximal effective concentration; E_max_, maximum effect; ERK, extracellular signal-regulated kinase; FC, fold change; IC_50_, half-maximal inhibitory concentration; MFI, mean fluorescence intensity; SEM, standard error of mean; WT, wild-type.

**
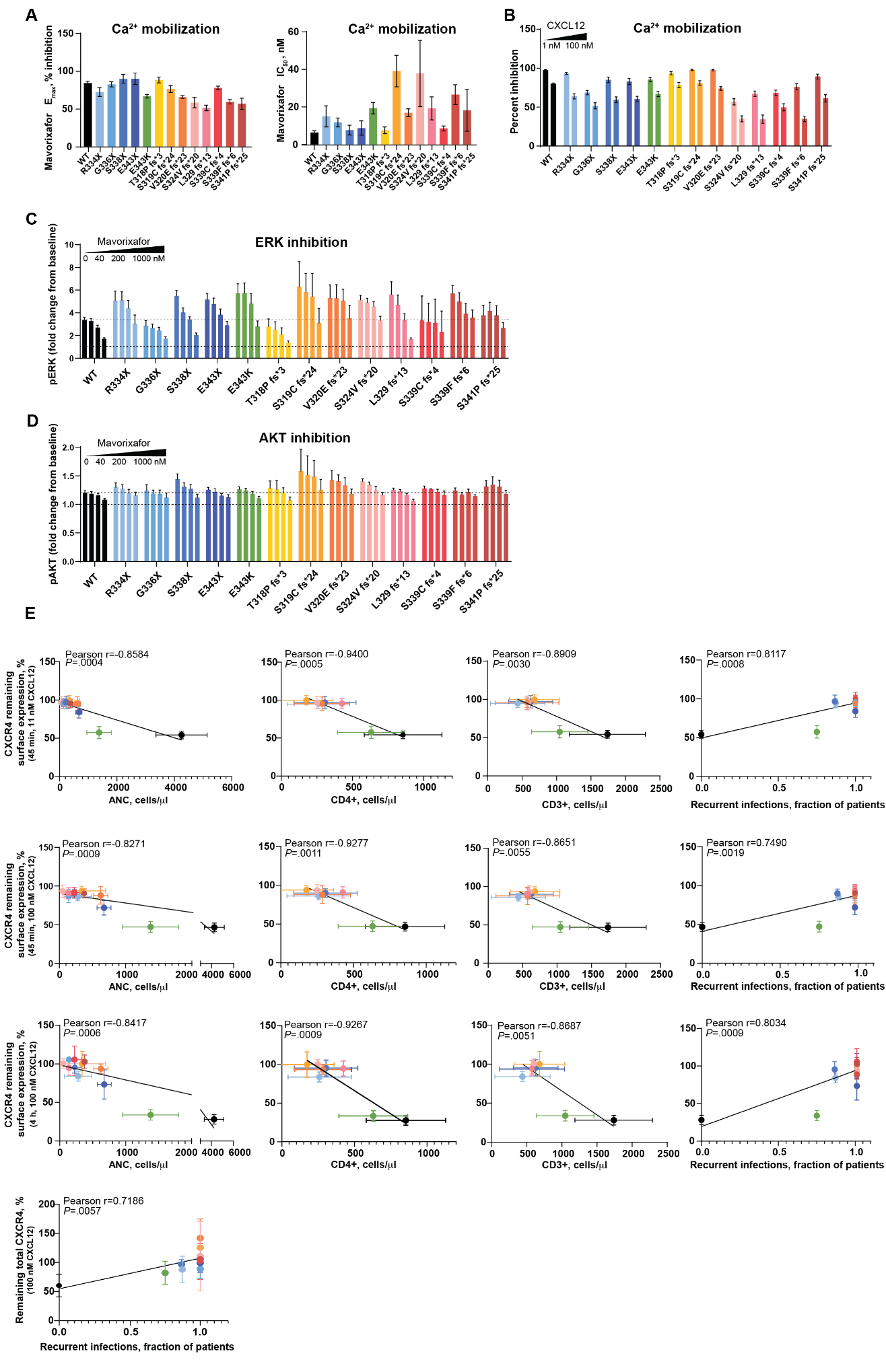
**
